# Heavy Metal Pollution Near a Tannery in Ulaanbaatar, Mongolia

**DOI:** 10.5696/2156-9614-7.16.2

**Published:** 2017-12-18

**Authors:** Erdenesaikhan Naidansuren, Altangerel Dondog, Batmunkh Erdenesaikhan, Enkhbayar Byambanyam

**Affiliations:** Environment and Security Center of Mongolia, Ulaanbaatar, Mongolia

**Keywords:** heavy metals, chromium, tanneries, Ulaanbaatar, Mongolia

## Abstract

**Background.:**

Due to an increase in population density and industrialization, the capital city of Mongolia, Ulaanbaatar, has been experiencing an increase in environmental pollution; especially soil contamination. Environmental pollutants include solid waste, silt and wastewater coming out of tanneries in three micro districts of the Khan-Uul district of Ulaanbaatar. Among the many types of chemicals these tanneries use, chromium (III) sulfate (Cr_2_(SO_4_)_3_) poses the most serious environmental health risk. In addition, the surrounding areas around the tanneries include schools, hospitals and residential buildings, presenting additional risks to the people living and working in this area.

**Objectives.:**

The present study aims to identify heavy metal contamination in the tannery area using a portable x-ray fluorescence reader (XRF).

**Methods.:**

The study area (112 ha) was divided into 6 zones depending on usage and XRF readings were taken in each zone.

**Results.:**

Results showed a mean or median lead (Pb) concentration of 2–405 mg/kg (33±2), chromium (Cr) concentration of 18–10,752 mg/kg (685±80), zinc (Zn) concentration of 5–1,316 mg/kg (113±6.5), and arsenic (As) concentration of 0–84 mg/kg (10.2±0.46) Five soil samples were collected from sites where Cr concentrations were extremely elevated and analyzed in two different laboratories to confirm XRF readings. Using the results from XRF readings and two different lab results, heavy metal distribution mapping was produced using geographic information system (GIS) tools, statistical processing tools and pollution indices for each heavy metal were determined using base heavy metals content in the soil. The distribution percentage of each of the heavy metals in the topsoil was 37.7% for Pb, 78.5% for Cr, 43.8% for Zn, and 51.3% for As.

**Discussion.:**

There are 140 tannery facilities in Mongolia of which 60 processing facilities reside in the Khan-Uul district of Ulaanbaatar. Tanneries use chromium (III) sulfate and other environmentally hazardous chemicals often in tandem with less costly technologies. This increases the amount of wastewater and contaminated silt going onto and into soil. The baseline level of heavy metals content was indicated by 7 XRF readings taken along the side of the Tuul river (relatively untouched soil). The present study shows that the Cr contamination in soil surrounding the tannery area was very high and the main source of the pollutants are wastewater and silt highly contaminated with chromium sulfate originating from the tanneries.

**Conclusions.:**

The present study found that the tannery area is heavily contaminated and may pose serious threats to human health, the surrounding environment and underground water resources. In order to reduce the health risk of the people working and living in the study area and remove contamination and rehabilitate the area, further studies are needed to determine heavy metal leakage into soil and underground water and to determine the volume of work needed for neutralization and rehabilitation.

## Introduction

Tanning is the process of treating skins and hides of animals to produce long lasting and durable leather. Traditionally, the process used tannin, an acidic chemical compound from which the tanning process draws its name. While there are many tanning agents, the use of a trivalent chromium (Cr (III)) solution was adopted by tanners during the Industrial Revolution and is still used extensively today in low- and middle-income countries.^1^ The Mongolian tannery sector was established in 1934 within the industrial complex in the Khan-Uul district. The transition in 1990 to a free market economy in Mongolia caused the tannery sector to decline. However, around 2003, private entities emerged, leading to a resurgence in the tannery sector, and there are currently approximately 140 tanneries in operation today.^2^

Chromium (III) sulfate, Cr_2_(SO_4_)_3_, is the most commonly used solution in these tanneries and primarily used during tanning, staining and surface processing. Even though it is moderately toxic, it is the common replacement for the more hazardous hexavalent chromium (Cr VI) process. Chromium (III) sulfate is granule, green in color, odorless and soluble in water. Occupational exposure to Cr (III), including chromium (III) sulfate may occur through inhalation of powdered raw material or through dermal contact with aqueous solution.

Inhalation of fine dust may cause apnea, headache, tinnitus, bronchitis, stomach pain, and shock and skin contact may cause burning, inflammation and allergy. Eye contact may cause permanent blindness. Exposure over a long period can cause nasal burning and lead to paracentesis.^3^ Dermal exposure to chromium (Cr) has been demonstrated to produce irritant and allergic contact dermatitis.^4^ The primary health effects of Cr (III) compounds occur in the human respiratory and immunological systems.^5^

In addition to Cr compounds, tannery effluent may contain a number of chemical and biological contaminants affecting human health such as aldehydes, alkalis, azo dyes, nitrophenols and various microbial parasites. Finally, the organic loading, i.e. biochemical oxygen demand, of the wastewater is very high and impacts receiving waters.^6^

Treatment plants in Ulaanbaatar are not designed to treat wastewater produced by these facilities and the tanneries do not neutralize their waste effluent. In addition, the wastewater sewage system was not built to handle the current organic load produced by tanneries. During high activity, these sewers overflow with water contaminated with Cr (III) and other agents. This overflow of highly contaminated water poses a risk of ground water contamination, as well as a respiratory risk from windswept dried deposited material. The particulates can also settle on crops and be carried into the residential human environment.

Several studies have investigated the environmental risk of Cr contamination in the surrounding area of the tanneries in Ulaanbaatar. These studies found that tanneries pose both a surface and groundwater contamination risk. A soil pollution study and eco-chemical assessment in Ulaanbaatar found that soil samples were highly contaminated with heavy metals, in particular Cr and lead (Pb) in the tannery sector.^7^,^8^ In addition, out of the 9 soil samples collected and analyzed using flame absorption atomic spectroscopy (AAS) at the soil laboratory of the Institute of Geography and Geoecology, Mongolian Academy of Sciences, 4 samples were found to have 1.2 to 27 times the precaution value set by the national standards Mongolian National Standard MNS 5850:2008 “Soil Quality. Soil Pollutants Elements and Substances”.^9^ Chromium-contaminated dust particles from the factories were carried by rain and wind, further contaminating the surrounding area's enveloping soil layer with Cr.^10^

**Abbreviations**
*PI*: Pollution index
*XRF*: X-ray fluorescence

Chromium found in soil most commonly exists in both the trivalent (Cr (III)) and hexavalent (Cr (VI)) forms. When Cr (III) resides in highly acidic soil with pH 2.7–4.5, the soil humin gets absorbed into the acid and becomes insoluble, immobile and inactive.^11^ Adsorption of Cr (VI) in neutral and alkaline (pH>6.8) soil resides in a low to medium soluble state (ex. Sodium chromate [Na_2_CrO_4_], barium chromate [BaCrO_4_], and lead (II) chromate [PbCrO_4_]).^12^ Finally, Cr (VI) is a known human carcinogen and has a high toxicity reference value.^13^

While the tannery industry has been studied in Ulaanbaatar, there is little data on metals aside from chromium in the vicinity of these sites. The purpose of the present study was to examine the extent of contamination and spatial distribution of heavy metals contamination in the soil in the area surrounding the tanneries. We tested the study area for Cr, Pb, arsenic (As), and zinc (Zn) contamination and the results of the study were disseminated to local government and representatives of the local residents.

## Methods

### Study Area

**Figure 1 —:**
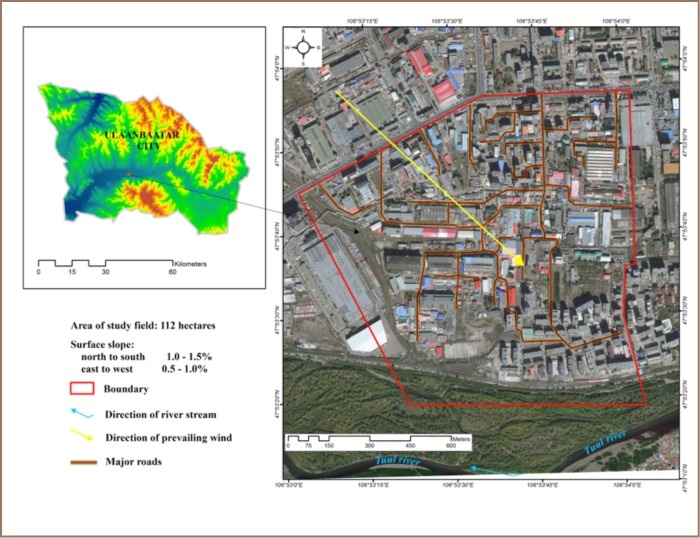
Study Area

The study area was located on approximately 112 hectares of land located along the Tuul river to the south of Ulaanbaatar city (E106.894, N47.894). The area is generally flat with a slight east-west and north-south slope at 1.0–1.5% (Figure 1). The soil base consists of alluvial gravel, sandy and silty loam, loose mealy moraine rocks and the enveloping layer is highly eroded and degraded.^14^ The groundwater level is 2.0–2.3 meters below the surface.^15^ The wind direction is generally from the northwest to southeast with an average wind speed of 4–6 m/s, with an average of 260 days a year with wind.^16^

**Figure 2 —:**
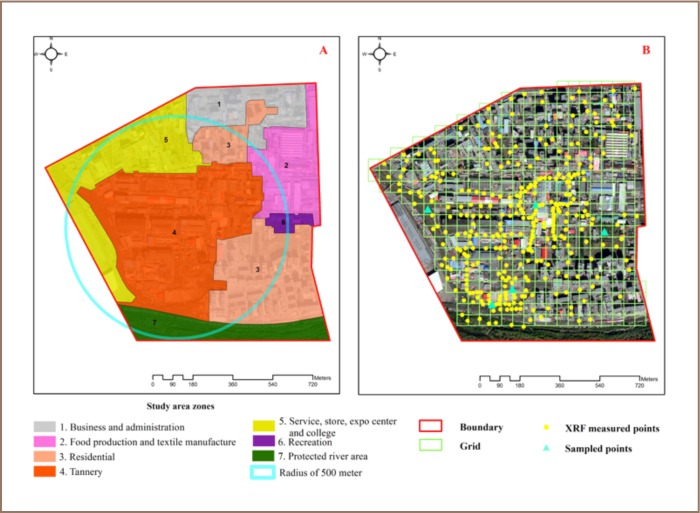
(A) Study area zones, (B) Locations of XRF readings within the study area

Approximately 10,000 people live and work in a 500 m radius around the tannery area.^17^ The area can be classified into 7 zones: 1) business and administration, 2) food production and textile manufacturing, 3) residential, 4) tannery, 5) service, stores, expo center and college, 6) recreation, and 7) a river protection zone (*Figure 2A*).

### Sampling Methodology

The study area was first surveyed then divided into 50 m × 50 m sampling grids. The soil was sampled on-site using a portable x-ray fluorescence reader (XRF) analyzer tool (Niton XL3t500) with daily pre- and post-calibration checks. Up to 2 samples (XRF readings) were taken within each grid cell. This was considered adequate given the uniformity of the sampling area. While every effort was made to ensure representative sampling, barriers such as fences, shrubbery, and structures periodically interfered with precise sampling locations. Wherever an XRF heavy metal reading exceeded the max allowable level in soil, as per Mongolian National Standard MNS5850:2008,^18^ we obtained additional readings within that cell to ensure data integrity. A total of 340 soil readings of heavy metals were taken (*Figure 2B*).

To ensure quality control and quality assurance, 5 topsoil samples were collected and submitted to Central Geological Laboratory of Mongolia and Société Générale de Surveillance laboratory (SGS Mongolia) for analytical confirmation. The quality control samples were taken from sampling grids containing elevated levels of heavy metals. The baseline level of heavy metals content was indicated by 7 XRF readings taken along the side of the Tuul river, which has relatively untouched soil.

### Laboratory Analysis

Heavy metals (Pb, Cr, Zn, As) in the soil samples were analyzed using laboratory-based XRF in two certified laboratories, the Central Geological Laboratory of Mongolia and the Société Générale de Surveillance laboratory (SGS Mongolia). Both laboratories prepared the soil samples for analysis by drying, weighing (lab reference: WGH79),^19^ milling, then homogenizing (lab reference: PUL46: Ringmill < 500 g sample, 90% passing 75 μm)^19^ the samples and measuring using a previously described method (lab reference: ICP40B: four-acid digestion/inductively coupled plasmaoptical emission spectrometry).^19^ The remaining homogenized soil samples received from the laboratories were measured by portable XRF analyzers to compare the differences in ground and non-ground samples. Finally, soil pH was determined using a potentiometric analyzer.

Soil sample pH was analyzed in the lab to examine the potential of solubility and leaching of heavy metals and their possible interactions with soil macro-elements such as manganese (Mn), potassium (K) and calcium (Ca).

### Statistical Analysis

All data were entered into a spreadsheet with appropriate variables and analyzed using XLSTAT (Addinsoft, Paris, France).^20^ The following tests were applied: descriptive statistics, correlation tests, Kolmogorov-Smirnov test, scatter plot (2D, y-axis values represent heavy metals content with automatic interval and x-axis values represent XRF reading indexes [1–340] at an interval of 50), and histogram (Data type → Continuous, Interval → 10, missing data → do not accept missing data, Histograms → Bars, Ordinate of the histograms → Frequency, Display a distribution → Normal [Standard]).

### Pollution Index

Each heavy metal in soil was measured and assessed using the single factor pollution index of Nomerov:^21^

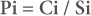
where Pi = pollution index, Ci = measured value, and Si = base value.

The purpose of choosing a single factor pollution index is to make clear which pollutant (heavy metal) exists in the study area and to what extent the area is polluted.

The pollution index is then divided into 4 classes in terms of degree of pollution severity (*Table 1*).

**Table 1 —:**
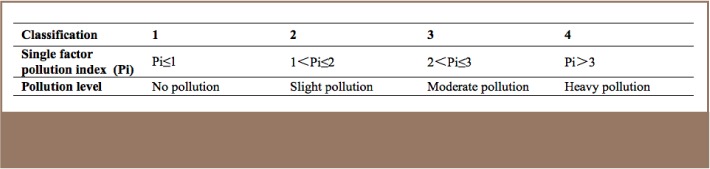
Single Factor Index Classification

### Imaging

We used conceptual imaging as a forecast of the spatial distribution of each heavy metal location interpolated using inverse distance weighting (Power-6, type standard, maximum neighbors-2, sector type-1, sector angle-0) with geo-statistical analyst tools in ArcGIS 10.2 (EA Desktop, Redlands, CA).

## Results

Soil pH Value and Macroelements (Manganese, Potassium and Calcium) Correlation coefficients between the 4 analytical methods (XRF-1, XRF-2, Lab-1 and Lab-2) showed strong correlation ranging from r = 0.87 to 1.00 and having a P value <0.01. The pH value of the five samples collected ranged between 7.69–8.30; slightly alkaline. The XRF soil readings showed ranges of 0.002–7.43% for Mn, 0.19–2.81% for K and 0.66–23.9% for Ca. As shown in Table 2, these macroelements showed no correlation with heavy metals.

**Table 2 —:**
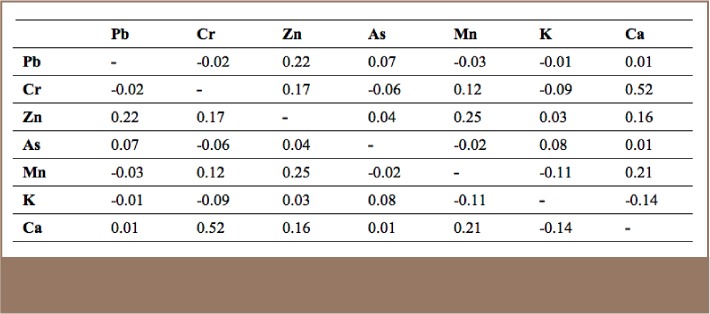
Correlation of Soil Macroelements

### Soil Heavy Metal (Lead, Chromium, Zinc, Arsenic) Content and Pollution Index

The present study considered only elements exceeding the precaution value indicated in the Mongolian National Standard MNS5850:2008 Soil Quality Max Allowable Level of Soil Contaminating Substances and Elements (*Table 3*).^18^ The pollution index of each element was defined using the base concentration value indicated in Table 3. The baseline level of heavy metals content was indicated by 7 XRF readings taken along the side of the Tuul river (relatively untouched soil). These baseline concentration values (i.e. background level) are the average concentration value in healthy soil without external influence.

**Table 3 —:**
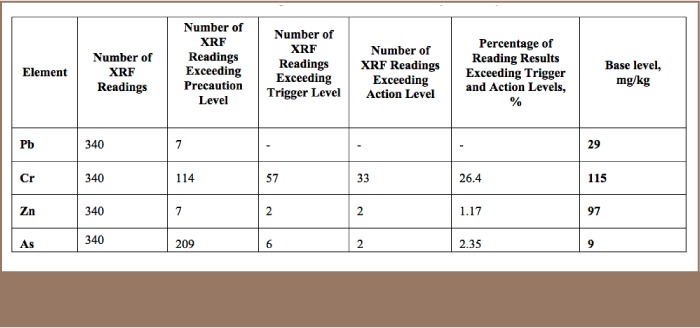
XRF Reading Results and Base Levels for Heavy Metals

XRF readings on 340 points revealed content level for Pb ranges (*Table 4*) [2–405.2 mg/kg (33±2)], Cr [18–10,752 mg/kg (685±80)], Zn [5–1,316 mg/kg (113±6.5)], and As ranges between [0–84 mg/kg (10.2±0.46)]. The study area consists of a total of 112 ha with a 55 ha area consisting of soil with vegetation cover, excluding areas under paved roads and buildings. In order to assess the spatial distribution of each heavy metal, geo-statistical analyst tools were deployed to interpolate the value of each contaminant linked with coordinates using inverse distance weighting. The study found that 37.7% of the 55 ha soil covered area is contaminated with Pb, 78.5% is contaminated with Cr, 43.8% with Zn and 51.3% by As.

**Table 4 —:**
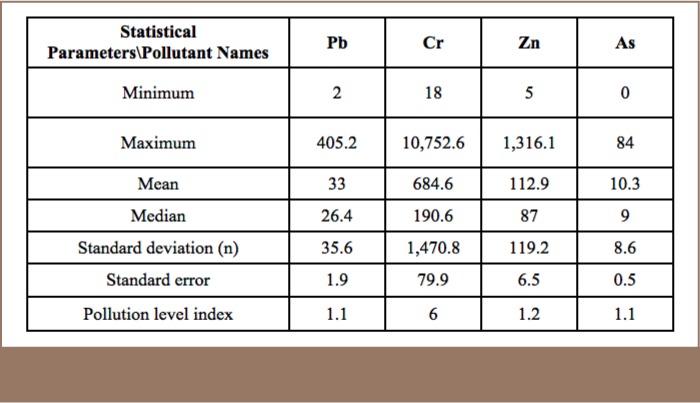
Statistical Characteristics and Pollution Level Index of Heavy Metals (mg/kg)

As presented in Table 5, the prevailing heavy metal was, as expected, Cr. However, other pollutants such as Pb, Zn, and As were present in the study area as well.

**Table 5 —:**
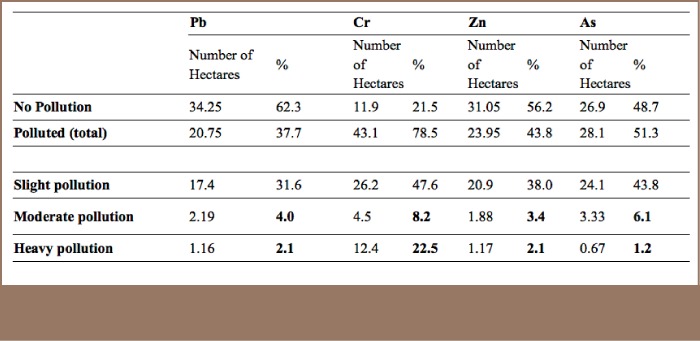
Spatial Distribution of Contaminants in 55 Hectare Soil Covered Study Area

### Lead

**Figure 3 (A, B) —:**
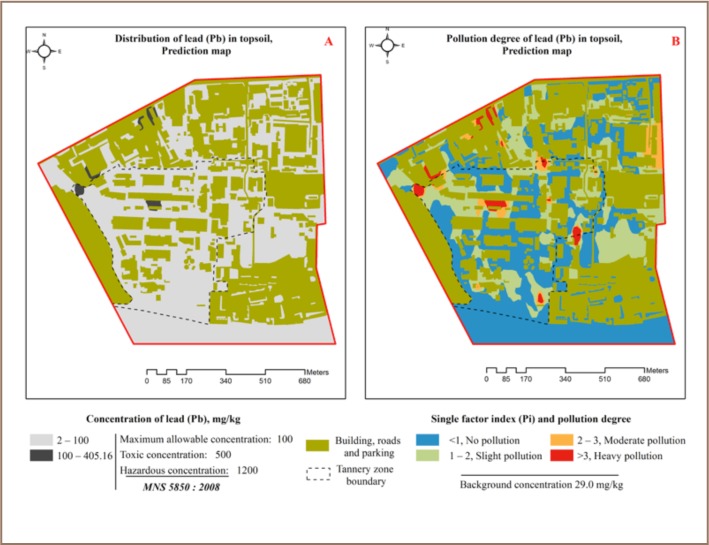
Lead distribution in topsoil (0–5 cm) and pollution index in the study area

As XRF reading results show, Pb concentrations in seven locations (2.1% of total measured Pb concentration) exceeded the precautionary value (*Table 3*). Five of these sampling points were within the tannery zone (*Figure 2*) and two were in the service, store, expo center and college zones. Lead contamination was relatively low in the study area and mostly originated from industrial solid and liquid waste openly disposed of outside. The samples with the highest Pb concentration were in the service, store, expo center and college zones, while Pb content in the river protection zone was the lowest. The highest concentration of Pb was 4 times higher than the precaution value. There was no correlation between other heavy metals and macro-elements and the correlation coefficient was under 0.25 (*Table 2*). In terms of pollution level index, 31.6% of the XRF readings indicated slight pollution, 6.1% indicated moderate to heavy pollution (*Table 5*), and the average pollution index was estimated to be 1.1. The spatial distribution of Pb in topsoil is shown in Figure 3 (A and B).

### Chromium

**Figure 4 (A, B) —:**
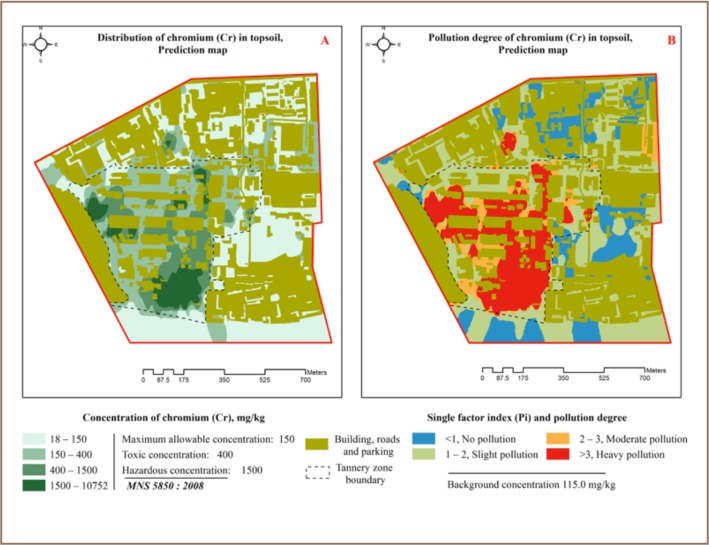
Chromium distribution in topsoil and pollution index

A total of 204 (60% out of 340) XRF readings of total Cr exceeded all standard set values and 114 (33.5%) readings exceeded the precaution value, 57 (16.8%) exceeded the trigger value and 33 (9.7%) readings exceeded the action value (*Table 3*). A total of 184 (88%) of these samples were in the tannery zone (*Figure 2*) and 20 (12%) came from other areas. The maximum concentration of Cr in the present study was 71 times higher than the precaution value (max allowable level or precaution value of 150 mg/kg according to the Mongolian National Standard on soil).^18^ The study results showed that the tannery area soil was highly polluted with Cr. The highest concentration of Cr (*Table 4*) was found in the tannery zone and the lowest concentration was found in the business and administration zone. The correlation coefficient was lower than 0.25, indicating no significant correlation between other heavy metals and the macro-elements K and Mn (*Table 2*), except for Ca with a correlation of 0.52. Soil Cr has the potential to change to calcium chromate (CaCrO_4_). According to the pollution index, 47.6% of the XRF reading results indicated slight pollution and 30.7% of the XRF reading results indicated moderate to heavy pollution (*Table 5*), with an average pollution index of 6.0. The spatial distribution of Cr (total) and its pollution index in topsoil in the study area are shown in Figure 4 (A, B).

### Zinc

**Figure 5 (A, B) —:**
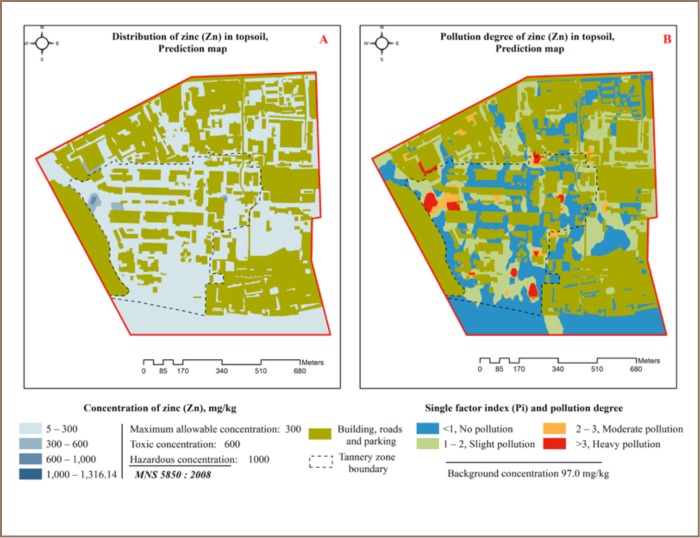
Zinc distribution in topsoil and pollution index

A total of 11 (4%) XRF readings showed Zn concentrations which exceeded the precaution value (*Table 3*). Of these, 9 (81.1%) were in the tannery zone (*Figure 2*) and 2 (18.9%) were in the service, store, expo center and college zone. Zinc contamination was relatively low in the study area and mostly originated from residential solid and liquid waste openly disposed of outside. The highest Zn concentration was located in the tannery zone and was 4.3 times higher than the precaution value. The correlation ratio with the other heavy metals and the macro-elements K and Ca was lower than 0.25, indicating no correlation (*Table 2*), except for Mn with a correlation value of 0.25 (very weak). According to the pollution index, 38% of the XRF readings indicated slight pollution and 5.5% indicated moderate to heavy pollution (*Table 5*), with an average pollution index of 1.2. The spatial distribution of Zn and Zn pollution in topsoil in the study area are shown in Figure 5 (A, B).

### Arsenic

**Figure 6 (A, B) —:**
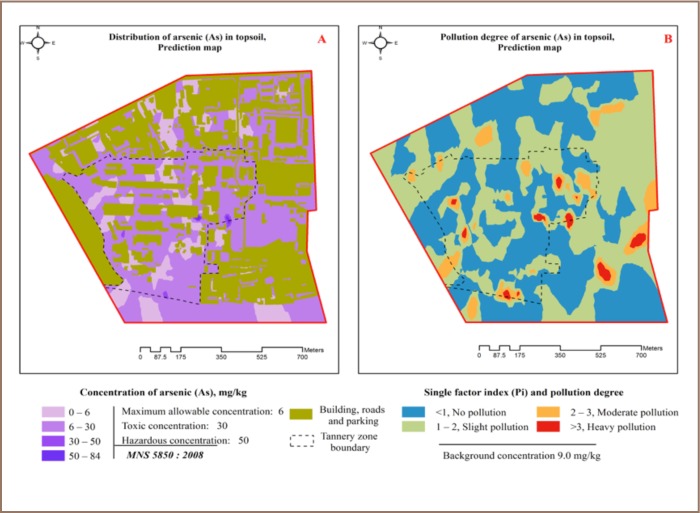
Arsenic distribution in topsoil and pollution index

A total of 217 (63.8%) of 340 XRF readings showed that As concentrations exceeded all standard set values (*Table 3*), and As was evenly distributed in all zones (*Figure 2*). A total of 209 (61.5%) readings indicated that the As content had exceeded the precaution value and 8 readings exceeded the trigger and action values. Arsenic is commonly found in the Ulaanbaatar soil surface and As contamination does not originate from tanneries, but from the underground alluvial rocks. The highest As concentration was found in the tannery and business and administration zones (*Table 4*). The correlation ratio with the other heavy metals and macro-elements was lower than 0.25, indicating no correlation (*Table 2*). According to the pollution index, 43.8% of the XRF readings indicated slight pollution and 7.3% indicated moderate to heavy pollution (*Table 5*), with an average pollution index of 1.1. The spatial distribution of As in topsoil in the study area is shown in Figure 6 (A, B).

### Comparison of Field Based Portable XRF Testing and Laboratory Analytical Results

**Figure 7 —:**
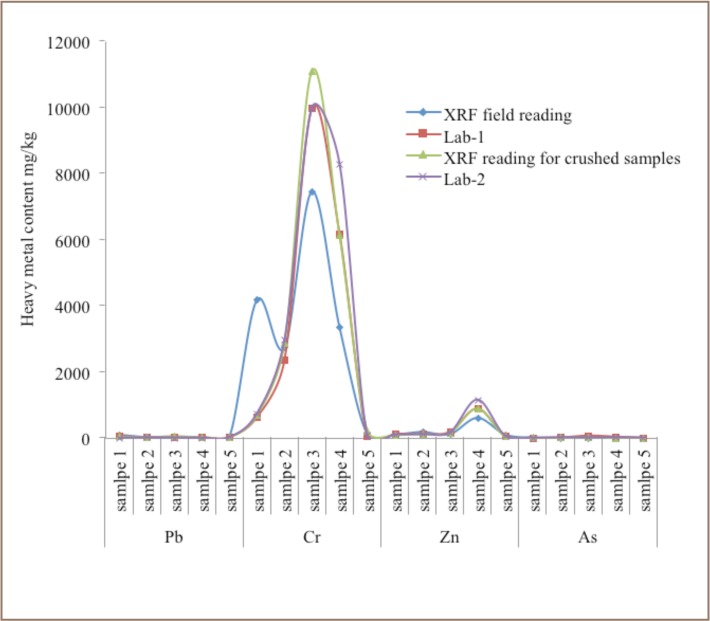
Comparison of XRF readings and sampled laboratory results

Five samples with high heavy metal values as measured by XRF readings were subsequently processed by Central Laboratory for Geology and Société Générale de Surveillance laboratory (SGS Mongolia) to confirm the accuracy of the XRF readings obtained in the field. The samples were milled to 0.075 mm before analysis. After obtaining the laboratory results, the samples were returned and re-tested using a portable XRF. Laboratory and field XRF readings were highly correlated (*Table 2, Figure 7*). The only exception was sample 1 for Cr content, where the field XRF readings were 5 times higher than the results from the two laboratories. In addition, the XRF field reading for sample 3 was 0.25% lower than the three other measurements. The reason for the difference between the XRF reading on the ground compared to the other three measurements is that particles in the topsoil differ in size, causing the X–rays to scatter in different directions, and therefore X-ray readings differ from the actual content. Correlation coefficients between the 4 analytical methods (XRF-1, XRF-2, Lab-1 and Lab-2) showed strong correlation ranging from r = 0.87 to 1.00 and having a P value <0.01.

## Discussion

There are 140 tannery facilities in Mongolia of which 60 processing facilities reside in the Khan-Uul district of Ulaanbaatar city. Tanneries use chromium (III) sulfate and other environmentally hazardous chemicals often in tandem with less costly technologies. This increases the amount of wastewater and contaminated silt going onto and into soil. The baseline level of heavy metals content was indicated by 7 XRF readings taken along the side of the Tuul river (relatively untouched soil). The present study shows that the Cr contamination in soil surrounding the tannery area was very high and the main sources of the pollutants are chromium sulfate-contaminated wastewater and silt originating from the tanneries.

Given that the study area soil has alkaline properties, the Cr in the soil has a high probability of being oxidized to the hexavalent (Cr+6) form and posing an additional health risk. Lead and Zn contamination is relatively low and mostly originates from residential solid and liquid waste disposed of openly outside.

Previous studies have shown that As levels were higher than the precaution value set by the Mongolian standards in MNS 5850:200818 in Mongolian soil. According to these standards there are three threshold values: precaution, trigger and action values. Arsenic contamination is not associated with tanneries, but rather originates from the underground alluvial rock. In 2014, researchers at the Institute of Geology and Mineral Resources, Mongolia conducted a soil study in eastern Mongolia in a 98,000 km^2^ area.^18^ Their study revealed an As content in untouched, healthy soil at 19 mg/kg on average, with a range of 5–56 mg/kg. The study concluded that As levels in Mongolian soil are higher than the precaution value set by the Mongolian standards in MNS 5850:200818, especially in subsoil. The main source of As appears to be arsenopyrite in the soil compositing rocks.^22^

The heavy metals in the present study pose health risks to residents and workers around the tannery area through wind dispersion of fine particles during the dry season and the risk of groundwater pollution through rainfall runoff. Most of the residential buildings are located to the east of the tanneries, which is downwind, and poses a risk of contaminated dust particles polluting the residential buildings (interior and exterior). In addition, after a rainfall there is a risk of heavy metal contamination spreading to areas such as schools, hospitals and office buildings through normal pedestrian traffic. Tanneries and other processing facilities may need to be relocated and industrial water treatment facilities upgraded to address the wastewater coming from these operations. In addition, the sludge from wastewater treatment has not been neutralized properly since the establishment of tanneries in the 1950's.

It is also probable that the Tuul river surface may be contaminated by Cr and other tannery-associated wastes. The presence of food processing plants, restaurants and food markets in close proximity to the tanneries poses a high risk of human health issues. The growing population and establishment of new residential areas are creating additional stresses on human health from industrial pollution. These residential areas include schools and kindergartens, presenting an elevated risk to children.

It is hoped that the current study results will raise awareness of municipal decision makers, newly resettled inhabitants and industrial workers to create a mutual agreement for effective management of tannery contamination in nearby residential areas.

## Conclusions

The present survey, while limited in scope, is one of the first reports of heavy metals in and around the tanneries of Ulaanbaatar. The dominant heavy metal contamination involves Cr from tanneries as it is the main agent used for tanning leather. The study examined 55 ha of soil cover for heavy metals contamination through 340 XRF readings. The contaminated area was estimated at 12.4 ha (22.5% of total study area) and revealed Cr to be the key pollutant. The technology employed by the tanneries uses excessive amounts of Cr for processing, causing soil, water and air pollution. Given the simple processing and extensive use of the Cr tanning process both in Mongolia and low- and middle-income countries, it is unlikely that industrial process engineering changes are forthcoming. In the last 10 years, the establishment of residential areas, food processing plants, markets, restaurants, schools, colleges and kindergartens has increased the health risks to the 10,000 people living and working in the study area. While further detailed studies of both Cr migration and characterization of other hazardous constituents produced by Ulaanbaatar's tanneries are needed, the present study demonstrates the need to better manage these facilities in Mongolia and other low- and middle-income countries.

## References

[1] Rivela B, Moreira MT, Bornhardt C, Mendez R, Feijoo G. Life Cycle Assessment as a Tool for the Environmental Improvement of the Tannery Industry in Developing Countries. *Environ Sci Technol* [Internet] 2004 [cited 2017 November 28] 38 6 1901– 1909 Available from: http://pubs.acs.org/doi/full/10.1021/es034316t Subscription required to view. 10.1021/es034316t 15074705

[2] Davaasuren B, Battulga B, Ariuntuya D, Munkhdorj G. Technology level assessment and results (Mongolian leather industries). 2016 International Conference on Innovation and Entrepreneurship Development; 2016 May 12–13; Ulaanbaatar, Mongolia Mongolia: Management & Innovation; 2016.

[3] Nriagu JO, Nieboer E Production and uses of chromium. : Nriagu JO, Nieboer E, *Chromium in natural and human environments*. New York: Wiley; 1988 Mar. p 81– 104.

[4] Bruynzeel DP, Hennipman G, van Ketel WG. Irritant contact dermatitis and chrome-passivated metal. *Contact Dermatitis* [Internet]. 1988 9 [cited 2017 Nov 29]; 19 3: 175– 9. Available from: http://onlinelibrary.wiley.com/doi/10.1111/j.1600-0536.1988.tb02889.x/pdf Subscription required to view. 10.1111/j.1600-0536.1988.tb02889.x 2973393

[5] Toxicological profile for Chromium [Internet]. Atlanta, Georgia: Agency for Toxic Substances and Disease Registry; 2012 [cited 2017 Nov 29] 47 p Available from: https://www.atsdr.cdc.gov/toxprofiles/tp7-c2.pdf 24049864

[6] Bosnic M, Buljan J, Daniels RP. Pollutants in tannery effluents [Internet]. Vienna: United Nations Industrial Development Organization; 2000 8 9 [cited 2017 Nov 29] 26 p. Report No.: US/RAS/92/120. Available from: https://leatherpanel.org/sites/default/files/publications-attachments/pollutants_in_tannery_effluents.pdf

[7] Batkhishig O, Bolormaa T, Enkhbayar B, Ouynbat P, Nyamsambuu N, Nyamdavaa B, Ikhbayar D, Ganzorig U. [Soil pollution and mapping of Ulaanbaatar city]. Ulaanbaatar, Mongolia: The Institute of Geography and Geoecology, Mongolian Academy of Sciences; 2011 Mongolian.

[8] Batkhishig O, Nyamsambuu N, Bolormaa T, Baymbaa G, Ganzorig U, Ikhbayar D, [Soil Pollution and Eco-Geochemical Assessment in Ulaanbaatar City]. Ulaanbaatar, Mongolia: The Institute of Geography and Geoecology, Clean Air Fund, Ministry of Environment and Green Development 2013 Mongolian.

[9] Oyunbat P, Enkhmunkh B Heavy metals pollution around tanneries in Ulaanbaatar city. Ulaanbaatar, Mongolia: Khurel Togoot Science Conference Proceedings; 2015.

[10] Kotas J, Stasicka Z Chromium occurrence in the environment and methods of its speciation. *Environ Pollut* [Internet]. 2000 3 [cited 2017 Nov 29]; 107 3: 263– 83. Available from: https://doi.org/10.1016/S0269-7491(99)00168-2 Subscription required to view. 10.1016/s0269-7491(99)00168-2 15092973

[11] Walsh AR, O'Halloran J Chromium speciation in tannery effluents – II. Speciation in effluent and in a receiving estuary. *Water Res* [Internet]. 1996 10 [cited 2017 Nov 29]; 30 10: 2401– 12. Available from: https://doi.org/10.1016/0043-1354(96)00174-1 Subscription required to view.

[12] Bartlett R, James B Behavior of chromium in soils: III. Oxidation. *J Environ Qual* [Internet]. 1979 [cited 2017 Nov 29]; 8 1: 31– 5. Available from: https://dl.sciencesocieties.org/publications/jeq/abstracts/8/1/JEQ0080010031?access=0&view=pdf Subscription required to view.

[13] Chromium (VI) compounds [Internet]. Vol. 100C. Lyon, France: International Agency for Research on Cancer; 2012 [cited 2017 Nov 29] 22 p. Available from: http://monographs.iarc.fr/ENG/Monographs/vol100C/mono100C-9.pdf

[14] Batkhishig O., Nyamsambuu N, Enkhbayar B, Ganzorig U, Byambaa G, Ouynbat P.. [Soil map of Ulaanbaatar city]. Ulaanbaatar, Mongolia: The Institute of Geography and Geoecology; 2011 Mongolian.

[15] Jadambaa N, Grimmelmann W, Kampe A. Explanatory notes for the hydrogeological map of mongolia 1:1000000. Hannover, Germany: German Geological Survey BGR; 2003 50 p.

[16] [Overview of soil quality in Ulaanbaatar 2016.] Ulaanbaatar, Mongolia: Annual Meteorological Bulletin Information and Research Institute of Meteorology, Hydrology and Environment of Mongolia; 2016 Mongolian.

[17] Population Census of 1, 2, 3rd micro districts of Khan-Uul district, Ulaanbaatar. Ulaanbaatar, Mongolia: Statistics Department of Ulaanbaatar city; 2016.

[18] [Soil quality: soil pollutant elements and substance] [Internet]. Ulaanbaatar, Mongolia: Mongolian National Standard; 2008 [cited 2017 Nov 29] 6 p. Report No.: MNS 5850. Mongolian. Available from: http://estandard.gov.mn/index.php?module=search&cmd=searching&start=0&per_page=10&q=5850&key=2&case=bugd

[19] Analytical services [Internet]. Geneva, Switzerland: SGS; 2017 [cited 2017 Nov 29] 86 p. Available from: http://www.sgs.com/-/media/global/documents/brochures/sgs-analytical-guide.pdf

[20] XLSTAT [Internet]. 2014.5. New York: Addinsoft; 2014 [cited 2017 Nov 29]. Available from: https://www.xlstat.com Subscrption required to view.

[21] Wang LF, Bai YX, Gai SN. Single-factor and nemerow multi-factor index to assess heavy metals contamination in soils on railway side of Harbin-Suifenhe Railway in Northeastern China. *Applied Mech Materi* [Internet]. 2011 7 [cited 2017 Nov 29]; 71–78: 3033– 6. Available from: https://www.scientific.net/AMM.71-78.3033 Subscrption required to view.

[22] Batkhishig O, Nyamsambuu N, Baymbaa G, Ganzorig U, Enkhbayar B, Nyambayar P. [Study of Eastern Mongolian soil arsenic distribution and mapping]. *J Geogr*. 2015; 11 27: 9– 17. Mongolian

[23] Multi acid (4-acid) digestions [Internet]. Geneva, Switzerland: SGS; c2017 [cited 2017 Nov 29]. [about 4 screens]. Available from: http://www.sgs.com/en/mining/analytical-services/geochemistry/digestion-and-fusion/multi-acid-4acid-digestions

